# Epidemiological aspects of healthcase-associated enterococcal infections

**DOI:** 10.1186/1753-6561-5-S6-P20

**Published:** 2011-06-29

**Authors:** E Brusina

**Affiliations:** 1 Department of Epidemiology, Kemerovo State Medical Academy, Kemerovo, Russian Federation

## Introduction / objectives

Enterococci (E.) have become a significant nosocomial pathogen, and their epidemiology is largely obscured.

## Methods

Data were obtained by monitoring of 1946 cases of E. infections in 1983-2010 including 17 outbreaks with 172 patients. Isolates (1728) were tested for susceptibility to ampicillin, gentamycin and vancomycin, 31 epidemic isolates of *E.faecium* were investigated by MLST (Gdh, PstS, PurK and AtpA loci).

## Results

Before 2000 maximal incidence of E. infections in Kemerovo Region hospitals was 0,8/1000 patients and share of *E.spp.* among all pathogens of HAIs was <2%. During the past decade the incidence of E. infections increased 20-fold and mortality rate increased 5-fold. The *E.faecalis/E.faecium* ratio was 9:1, but in bacteremia cases *E.faecium* was predominant. Hospitalized patients had 63% isolation rate of *E.faecalis*, depending on duration of hospital stay. Primary selection of epidemic *E.spp*. clones occurred in ICU, further spreading to other units. The *E.spp* epidemic potential was as high as of *S.typhimurium* and *P.aeruginosa*. The level of epidemic hazard was higher for *E.faecium* compare to *E.faecalis*. The minimal selection time for epidemic clones of *E.spp*. was 7 days (maximal circulation-4 months). Specific allelotype of the purK1 gene was related to hospital outbreaks. Infections mainly arised from endogenous sources, mainly after colonization of the gastrointestinal tract by epidemic *E.spp*. clones, but infection has also spread from patient to patient or by hands of healthcare staff, uniform or indirectly by contaminated surfaces and equipment. About 40% of *E.faecalis* isolates and 72% of *E.faecium* isolates were resistant to ampicillin, 79% and 82% - to gentamycin, respectively. The VRE share was 31%. Epidemic clones of E. survived on surfaces in hospital environment for 37 days.

## Conclusion

Our findings can be taken into account for prevention of HAIs.

## Disclosure of interest

None declared.

